# Are Routinely Collected NHS Administrative Records Suitable for Endpoint Identification in Clinical Trials? Evidence from the West of Scotland Coronary Prevention Study

**DOI:** 10.1371/journal.pone.0075379

**Published:** 2013-09-13

**Authors:** Sarah J. E. Barry, Eleanor Dinnett, Sharon Kean, Allan Gaw, Ian Ford

**Affiliations:** 1 Robertson Centre for Biostatistics, University of Glasgow, Glasgow, United Kingdom; 2 R&D Department, Greater Glasgow and Clyde Health Board, Glasgow, United Kingdom; 3 Wellcome Trust/Wolfson Clinical Research Facility, Queen's University Belfast, Belfast, United Kingdom; Wake Forest University Health Sciences, United States of America

## Abstract

**Background:**

Routinely collected electronic patient records are already widely used in epidemiological research. In this work we investigated the potential for using them to identify endpoints in clinical trials.

**Methods:**

The events recorded in the West of Scotland Coronary Prevention Study (WOSCOPS), a large clinical trial of pravastatin in middle-aged hypercholesterolaemic men in the 1990s, were compared with those in the record-linked deaths and hospitalisations records routinely collected in Scotland.

**Results:**

We matched 99% of fatal study events by date. We showed excellent matching (97%) of the causes of fatal endpoint events and good matching (>80% for first events) of the causes of nonfatal endpoint events with a slightly lower rate of mismatching of record linkage than study events (19% of first study myocardial infarctions (MI) and 4% of first record linkage MIs not matched as MI). We also investigated the matching of non-endpoint events and showed a good level of matching, with >78% of first stroke/TIA events being matched as stroke/TIA. The primary reasons for mismatches were record linkage data recording readmissions for procedures or previous events, differences between the diagnoses in the routinely collected data and the conclusions of the clinical trial expert adjudication committee, events occurring outside Scotland and therefore being missed by record linkage data, miscoding of cardiac events in hospitalisations data as ‘unspecified chest pain’, some general miscoding in the record linkage data and some record linkage errors.

**Conclusions:**

We conclude that routinely collected data could be used for recording cardiovascular endpoints in clinical trials and would give very similar results to rigorously collected clinical trial data, in countries with unified health systems such as Scotland. The endpoint types would need to be carefully thought through and an expert endpoint adjudication committee should be involved.

## Introduction

There is increasing interest in the use of routinely collected health records to support clinical trials. In England, the Clinical Practice Research Datalink [1] and in Wales, the Secure Anonymised Information Linkage [2] initiative, support researchers in conducting studies. In Scotland, the Wellcome Trust funded Scottish Health Informatics Programme [3] has created a Scotland-wide research platform for the collation and analysis of electronic patient records. At the same time, there is a desire to reduce the complexity and cost of conducting clinical trials. In the United States, the Clinical Trials Transformation Initiative [4] has been established to drive forward changes in this area. In the United Kingdom, the Academy of Medical Sciences has put forward proposals for improvement of the research environment including increased use of routinely collected health data. Recently, the Medical Research Council and other major funders in the UK have created of a network of four health eResearch Centres as centres of excellence in research using e-health records.

The future success of the UK Biobank [5] project will depend in large part on achieving reliable follow-up for clinical outcomes at reasonable cost. The widely used Scottish Longitudinal Study [6] and the England & Wales (ONS) Longitudinal Study [7] are based on linking census data with routinely collected data such as deaths and hospital admissions, and have led to research in a wide range of areas, from health services research to employment (eg. [8], [9]). Routinely collected data has been linked to nurse/researcher-collected data in the Scottish Health Survey, to follow up individuals for outcomes that would be resource intensive to collect by other means [10], and such data has been used to highlight risk factors to the physical and mental health of the Scottish population [11], [12]. All of these databases are available for research, whether it be for public health or clinical trial follow-up.

An important question is whether routinely collected data, especially data collected for administrative reasons, are fit for purpose as outcomes in clinical trials or epidemiological studies.

In 2007 we were able to extend the original average follow-up of approximately 5 years in the West of Scotland Coronary Prevention Study (WOSCOPS) by approximately 10 years [13] using computer record linkage to nationally held datasets for hospital admission, incident cancers and deaths in Scotland. WOSCOPS was a randomised double-blind placebo-controlled trial of pravastatin in 6595 hypercholesterolaemic men aged 45–64 with no history of myocardial infarction, carried out between 1988 and 1995. The study achieved 100% follow-up of all participants for vital status at the end of the trial. The main endpoints of the trial included definite or suspect coronary heart disease (CHD) death, definite or suspect nonfatal myocardial infarction (MI), other cardiac death, other vascular death or other death. Further details on the design and conduct of the trial may be found elsewhere [14].

The results in the original report of the trial [15] were obtained via intensive and expensive follow-up of participants and detailed recording and adjudication of all clinical outcomes. In the current work we have carried out record linkage follow-up of outcomes during the trial period, and explored the pros and cons of record linkage versus intensive follow-up in the context of cardiovascular endpoints. This research adds detail to an initial assessment of the utility of record linkage data for general serious adverse event reporting [16] and shows that it could also be used to follow patients up on specific endpoints, particularly if an endpoint committee were involved.

## Methods

### Overview

Approximately 10 years after the end of the trial, WOSCOPS participants were probabilistically matched using name, date of birth and postcode to national datasets for mortality, incident cancer and hospital discharges by the Information Services Division (ISD) of the NHS National Services Scotland, providing computerised record linkage follow-up of all trial participants at low cost.

This paper compares and contrasts the events that were reported by the normal trial processes and the events that were identified by record linkage as occurring during the period of the trial. We focus on deaths, non-fatal MI and non-fatal stroke.

### Ethics Statement

The participants in WOSCOPS provided written informed consent for access to their medical records, but at the time this did not explicitly refer to medical records held on a computer. After the end of the trial, the ethics committee of the Royal Infirmary, Glasgow, approved linkage of patient information to the participants' electronic medical records. In addition the Privacy Advisory Committee of National Services Scotland, which acts as the gatekeeper to Scotland's national holdings of patient records and advises ISD, also approved this linkage. Both approvals are necessary before a linkage can be performed. These processes are long established in Scotland as the appropriate pathway for record linkage approvals. Hence, we have strictly adhered to all existing ethical and information governance requirements in Scotland. In addition all data were handled in compliance with the UK data protection regulations.

### WOSCOPS Trial Events

During the study, all deaths and all non-fatal coronary events were reviewed by an endpoint adjudication committee. Although not pre-specified as an endpoint, potential stroke or transient ischaemic attack (TIA) events were also documented on a stroke-specific form and were reviewed centrally at the end of the study and classified as stroke, TIA or ‘other’. Imaging was not always available; hence we have not further grouped these events as haemorrhagic or non-haemorraghic. The original WOSCOPS definition of a nonfatal MI included events that were ‘silent’ (only detected via an annual study electrocardiogram without accompanying symptoms) or ‘unrecognised’ (symptoms consistent with an MI detected but not recognised as an MI at the time). Since the events in the routinely collected dataset were, by definition, hospitalisations, we included only hospitalised study events in the analysis, excluding silent events but including unrecognised hospitalised events. Events originally classified as ‘definite’ or ‘suspect’ were merged. Nonfatal events considered plausibly an MI were adjudicated as either a nonfatal MI or as a nonfatal ‘other’ event.

### Record Linkage (RL) Events

Death records during the trial provided the date of death, an underlying cause of death and a total of up to four causes coded using the International Classification of Diseases (ICD) codes, version 9. For RL identified deaths, cause of death was taken to be the assigned underlying cause of death.

The Scottish Morbidity Records of hospitalisations contain the dates of admission and discharge from hospital, along with dates of ward transfers and up to six diagnoses for each ward stay, all ICD coded. For analyses involving non-fatal MI, stroke or transient ischaemic attack (TIA), a relevant code in any position of a hospitalisation report was taken as evidence of an event. Hemiplegia was included as a cerebrovascular (stroke/TIA) diagnosis in the RL dataset.

We searched in the RL dataset for all events recorded as MI. In the absence of MI, any code for CHD in any position was taken as evidence of a CHD-related event. In the absence of a CHD code, a cardiovascular (CV) code in any position identified a CV-related outcome, and in the absence of a CV code the event was considered non-cardiovascular. The ICD code groupings for events identified by RL are given in Appendix S1.

### Statistical Methods

WOSCOPS and RL events for an individual were considered to be the same event if they had a diagnosis match and the event dates were within at most two weeks of one another. A detailed study of any WOSCOPS events unmatched in the RL data was carried out using the patient casenotes. ‘First events’ were defined as first events of the particular type (eg. MI) during the study period.

The analysis of the original WOSCOPS events was carried out for the fatal and hospitalised nonfatal WOSCOPS events, and similarly for the RL events. These analyses used the log-rank test to calculate the p-values and Cox proportional hazards models to produce the risk reduction estimates and corresponding 95% confidence intervals.

## Results

The baseline characteristics of the participants in the WOSCOPS are given in Table 1. Participants were on average 55 years of age and had mean baseline total cholesterol and low density lipoprotein cholesterol levels of 7.0 and 5.0 mmol/l respectively.

**Table 1:** Baseline characteristics.

Characteristic	Placebo (N = 3293)	Pravastatin (N = 3302)
**Continuous variables (mean (SD))**		
Age (years)	55.1 (5.5)	55.3 (5.5)
Body Mass Index (kg/m^2^)	26.0 (3.1)	26.0 (3.2)
Systolic Blood Pressure (mmHg)	136 (17)	135 (18)
Diastolic Blood Pressure (mmHg)	84 (10)	84 (11)
Total cholesterol (mmol/l)	7.0 (0.6)	7.0 (0.6)
LDL cholesterol (mmol/l)	5.0 (0.4)	5.0 (0.4)
HDL cholesterol (mmol/l)	1.1 (0.3)	1.1 (0.2)
Triglycerides (mmol/l)	1.85 (0.77)	1.83 (0.79)
**Categorical variables (n (%))**		
+ve Rose Questionnaire (angina)	174 (5%)	164 (5%)
+ve Rose Questionnaire (claudication)	96 (3%)	97 (3%)
Diabetes mellitus	35 (1%)	41 (1%)
Hypertension	506 (15%)	531 (16%)
Current smoker	1460 (44%)	1445 (44%)
Former Smoker	1127 (34%)	1138 (34%)
Never smoker	705 (21%)	717 (22%)

Baseline characteristics of the WOSCOPS population split by treatment group.

The main original results of the WOSCOPS are given in Table 2, alongside results excluding non-hospitalised MI and those obtained using RL outcomes. While the observed risk reduction in the primary endpoint of CHD death or nonfatal MI was slightly greater using the RL data than the original or hospitalised WOSCOPS data, the qualitative conclusion would have been the same regardless of the approach. A similar impact is observed for nonfatal MI alone, while the risk reduction for CHD death is slightly reduced, going from being marginally significant using the original or hospitalised WOSCOPS events to marginally nonsignificant using the RL events.

**Table 2:** Original WOSCOPS results and corresponding results using hospitalised WOSCOPS and Record Linkage events.

	Original WOSCOPS events				Hospitalised WOSCOPS events				Record Linkage events			
Endpoint type	Placebo(n = 3293)	Pravastatin(n = 3302)	P	%RR(95% CI)	Placebo(n = 3293)	Pravastatin(n = 3302)	P	%RR(95% CI)	Placebo(n = 3293)	Pravastatin(n = 3302)	P	%RR(95% CI)
CHD death or MI	295 (9.3)	215 (6.5)	<0.001	29 (15, 44)	212 (6.7)	147 (4.6)	<0.001	32 (16,45)	195 (6.1)	121 (3.8)	<0.001	39 (24,51)
Nonfatal MI	246 (7.8)	182 (5.8%)	0.001	27 (12, 40)	156 (4.9)	112 (3.6)	0.005	29 (10,45)	143 (4.5)	87 (2.7)	<0.001	40 (22,54)
CHD death	61 (1.9)	41 (1.3)	0.042	33 (1,55)	–	–	–	–	56 (1.7)	40 (1.3)	0.093	29 (-6,53)

Original results from the WOSCOPS study (combining definite and suspect coronary events) compared with those omitting non-hospitalised myocardial infarction (MI) and with results based entirely on record linkage. Data shown are numbers and rates (%) of events for each treatment group, p-value and % risk reduction (RR) for the outcomes of coronary heart disease (CHD) death or MI, non fatal MI and CHD death.

### Fatal Events

There were 241 deaths recorded in WOSCOPS and 240 in the RL dataset. Of these 238 were matched by date. Three WOSCOPS deaths were missing from the RL dataset and further investigation showed that they all occurred outside Scotland (1 Bulgaria, 2 England). Two deaths were found in the RL dataset but not recorded in WOSCOPS and these appeared to be errors in the linkage, since one had a date of death prior to the participant’s randomisation date and thus could not be the same person, while the other had no recorded events in WOSCOPS.

The primary causes of death for the 238 deaths that matched are given in Table 3. The RL data distinguished between 29 ‘other’ and 91 ‘cancer’ deaths (which are all denoted as ‘other’ in the table), with 2 of the ‘other’ deaths being mismatched as CHD and 1 cancer death as ‘other vascular’ in WOSCOPS. There was a high rate of matching on cause of death, with only 7/238 (2.9%) of causes being mismatched. A clinical review of the death certificates for these 7 patients, blinded to the WOSCOPS outcome, re-classified 4 of the events, reducing the mismatch rate to 3/238 (1%).

**Table 3:** Matching of the causes of death between WOSCOPS and Record Linkage.

	Record Linkage cause of death					
WOSCOPS causeof death	CHD	Other cardiac	Other vascular	Other		Total
**CHD**	96	1	1	2		100
**Other cardiac**	0	3	0	0		3
**Other vascular**	0	2	15	1		18
**Other**	0	0	0	117		117
**Total**	96	6	16	120		238

Comparison of WOSCOPS death classification with Record Linkage classification in the 238 deaths identified by both approaches. ‘Other’ and ‘cancer’ Record Linkage deaths have been combined into a single ‘other’ category.

### Nonfatal Events

#### The distribution of RL events conditional on WOSCOPS MI events

There were 304 cases of nonfatal MI incurring hospitalisation in WOSCOPS, 268 of which were first hospitalised MI events. Table 4 shows the distributions of corresponding RL events for first and subsequent WOSCOPS MI events.

**Table 4:** Matching of WOSCOPS first and subsequent MI events with corresponding Record Linkage events.

	Record Linkage events							
	1^st^ MI	Subsequent MI	CHD	CVD	CP	Other	Non-match	Total
**First WOSCOPS MI event**	217 (81.0%)	1 (0.4%)	22 (8.2%)	3 (1.1%)	15 (5.6%)	2 (0.7%)	8 (3.0%)	268
**Subsequent WOSCOPS event**	4 (11.1%)	17 (47.2%)	12 (33.3%)	0 (0.0%)	2 (5.6%)	0 (0.0%)	1 (2.8%)	36
**Total**	221 (72.7%)	18 (5.9%)	34 (11.2%)	3 (1.0%)	17 (5.6%)	2 (0.6%)	9 (3.0%)	304

WOSCOPS first and subsequent MI events matched with Record Linkage events by cause and date (MI = myocardial infarction; CHD = Coronary Heart Disease; CVD = other cardiovascular disease; CP = non-cardiac chest pain). ‘Other’ matches are for a 'date matched' event with a diagnosis other than MI, CHD, CVD or CP and non-matches have no near date match.

The matching of MI events is better for first than subsequent events, with 81.4% of WOSCOPS first MI events versus 58.3% of subsequent MI events matching with MI events in RL. The classification rate of WOSCOPS MI events as CHD events (including MI) is similar for first and subsequent events, however, with 89.6% and 91.6% concordance, respectively. Of particular note is that over half of the WOSCOPS MI events not matched with cardiovascular events in RL (15/25 of first and 2/3 of subsequent nonmatched events) were matched with RL events diagnosed as non-cardiac ‘unspecified chest pain’, suggesting that there may be a number of miscodings for such events in hospital records. Of the 9 WOSCOPS MI events that were not found at all in the RL dataset, 7 of the events occurred outside Scotland, 1 patient was admitted to a private hospital (only NHS admissions are recorded in the hospitalisations dataset) and 1 was definitely admitted to hospital according to the casenotes and their absence from the hospitalisations dataset is presumed due to linkage errors.

#### The distribution of WOSCOPS MI events conditional on RL MI events

Of the 264 hospitalisations identified in RL as MI, 230 were first events, and 96.0% of these were matched with a WOSCOPS MI event (Table 5). A total of 7 (3.0%) first RL MI events were matched with ‘other’ events in WOSCOPS, all of which had been adjudicated by the WOSCOPS adjudication committee as not being MI events. Of the 10 (29.4%) subsequent events matched with ‘other’ events in WOSCOPS, 9 were similarly adjudicated as not being MI events, while one had MI as the secondary diagnosis to a primary diagnosis of chest pain and the patient casenotes showed it involved a procedure following an earlier (matched) MI and was therefore not a ‘true’ MI admission. Of the 2 (0.9%) first event hospitalisations with an MI diagnosis that were not matched with WOSCOPS MI endpoint events, the patient casenotes showed that one was a day admission for a procedure and the other was admitted with atrial fibrillation and had been confirmed to the trial committee by the patient’s cardiologist as having no evidence of MI. Neither event, therefore, was referred to the endpoint committee.

**Table 5:** Matching of Record Linkage first and subsequent MI events with corresponding WOSCOPS events.

	WOSCOPS events				
	1^st^ MI	Subsequent MI	Other	Non-match	Total
**First RL MI event**	217 (94.3%)	4 (1.7%)	7 (0.3%)	2 (0.9%)	230
**Subsequent RL event**	1 (2.9%)	17 (50.0%)	10 (29.4%)	6 (17.6%)	34
**Total**	218 (82.6%)	21 (8.0%)	17 (6.4%)	8 (3.0%)	264

Record Linkage first and subsequent MI events matched with WOSCOPS events. ‘Other’ matches correspond to a date match with events adjudicated by the adjudication committee as not being MI, and non-matches had no near date match.

Of the 6 (17.6%) subsequent event hospitalisations with an MI diagnosis that were not matched with WOSCOPS MI endpoint events, the patient casenotes show that 3 were readmissions following matched MI events (one involving a procedure), 1 was a transfer from a private hospital following an earlier admission for a MI (and therefore not a true admission), 1 was admitted to Accident & Emergency only and diagnosed with heart failure and 1 was admitted for pulmonary oedema. None of these events were referred to the endpoint committee.

#### Distribution of RL events conditional on a WOSCOPS stroke or TIA

There were a total of 94 WOSCOPS events for stroke or TIA, 83 of which were first stroke/TIA events, and the level of matching with RL events is shown in Table 6. Of the WOSCOPS first stroke events, 78.7% were matched with either RL stroke (72.1%) or TIA hospitalisations (6.6%). For WOSCOPS first TIA events, the match rate was similar at 77.2% (54.5% matched as TIA, 22.7% as stroke).

**Table 6:** Matching of the WOSCOPS strokes and TIAs.

	Record Linkage events					
First WOSCOPS event	Stroke	TIA	MI	Other	Non-match	Total
**Stroke**	44 (72.1%)	4 (6.6%)	1 (1.6%)	4 (6.6%)	8 (13.1%)	61
**TIA**	5 (22.7%)	12 (54.5%)	1 (4.5%)	0 (0.0%)	4 (18.2%)	22
**Total**	49 (59.0%)	16 (19.3%)	2 (2.4%)	4 (4.8%)	12 (14.5%)	83
	**Record Linkage events**					
**Subsequent WOSCOPS event**	**Stroke**	**TIA**	**MI**	**Other**	**Non-match**	**Total**
**Stroke**	10 (100.0%)	0 (0.0%)	0 (0.0%)	0 (0.0%)	0 (0.0%)	10
**TIA**	0 (0.0%)	1 (100.0%)	0 (0.0%)	0 (0.0%)	0 (0.0%)	1
**Total**	10 (90.9%)	1 (9.1%)	0 (0.0%)	0 (0.0%)	0 (0.0%)	11

Matching of WOSCOPS first and subsequent strokes and transient ischaemic attacks (TIA) by cause and date with Record Linkage events. The ‘other’ RL events were any hospitalizations with a date match but a diagnosis other than stroke, TIA or MI and non-matches had no near date match. A first event was defined as *either* a stroke *or* a TIA event and could match with a first or subsequent event of the opposing type.

Of the WOSCOPS stroke/TIA first events, 12 were completely unmatched, of which 6 occurred outside Scotland, 1 was an admission to a private hospital, 1 was missing supporting documentation to confirm their admission date, 3 had definite admissions with stroke/TIA diagnoses, suggesting a possible problem with RL, coding or admission dates and 1 had unspecified cerebrovascular disease. There were only 11 instances of subsequent WOSCOPS stroke/TIA events, all of which were matched in RL.

#### Distribution of WOSCOPS events conditional on a RL stroke or TIA

Table 7 shows that there were a total of 102 hospitalisations in the RL data for stroke or TIA events, 87 of which were first events. Of the RL first stroke events, 83.1% were matched with either WOSCOPS stroke (75.4%) or TIA (7.7%) events. For RL first TIA events, the matching rate was slightly lower at 72.7% (54.5% as TIA, 18.2% as stroke). There was a higher level of mismatching of a small number of subsequent stroke/TIA events.

**Table 7:** Matching of the Record Linkage strokes and TIAs.

	WOSCOPS event					
First Record Linkage event	Stroke	TIA	MI	Other	Non-match	Total
**Stroke**	49 (75.4%)	5 (7.7%)	1 (1.5%)	2 (3.1%)	8 (12.3%)	65
**TIA**	4 (18.2%)	12 (54.5%)	1 (4.8%)	1 (4.5%)	4 (18.2%)	22
**Total**	53 (60.9%)	17 (19.5%)	2 (2.3%)	3 (3.4%)	12 (13.8%)	87
	**WOSCOPS event**					
**Subsequent Record Linkage event**	**Stroke**	**TIA**	**MI**	**Other**	**Non-match**	**Total**
**Stroke**	5 (38.5%)	0 (0.0%)	0 (0.0%)	1 (7.7%)	7 (53.8%)	13
**TIA**	0 (0.0%)	1 (50.0%)	0 (0.0%)	0 (0.0%)	1 (50.0%)	2
**Total**	5 (33.3%)	1 (6.7%)	0 (0.0%)	1 (6.7%)	8 (53.3%)	15

Matching of Record Linkage events for stroke and TIA with WOSCOPS events. The ‘other’ WOSCOPS events were date matches that were originally recorded as MI in WOSCOPS but were adjudicated by the expert committee as not being an MI, and non-matches had no near date match. A first event was defined as *either* a stroke *or* a TIA event and could match with a first or subsequent event of the opposing type.

The RL stroke/TIA events that were matched with MI endpoints had both MI and stroke/TIA diagnoses for the same hospitalisation. Of the 15 ‘other’ matches and the non-matches for RL first stroke or TIA events in record linkage, 6 were capturing a pre-existing condition according to the patient notes, 2 were capturing a cerebral event other than stroke/TIA, 4 had unclear diagnosis or the diagnosis was clarified later, 1 was not an acute event and 2 were unexplained and may have been due to inaccurate coding or additional information in the medical record. Of the 9 ‘other’ matches and the non-matches for RL subsequent stroke or TIA events, 8 were capturing a pre-existing condition, while for 1 the event was recorded on the case report forms in WOSCOPS but the specific stroke/TIA form was not completed.

## Discussion

It is clear that, if WOSCOPS had only considered fatal events and nonfatal events that incurred hospitalisation, the researchers would have drawn very similar conclusions using record linkage data only compared to using adjudicated outcomes. We have shown, for cardiovascular data collected in Scotland, good matching of routinely collected and rigorously collected clinical trial data, in particular for mortality. The matching was also good for MI endpoints, particularly for first events. The quality of matching was less good for the stroke and TIA non-endpoint events.

Due to the imperfect matching of events, our proposed model of conducting a cardiovascular clinical trial using RL data in a unified healthcare system would be to use RL for case finding and to have an expert endpoint committee that would adjudicate based on appropriate source documentation from the patient’s clinical record. Based on our results we believe that this model would be likely to detect most of the MI events that were coded in the hospitalisations dataset as CHD or cardiovascular events. It would also be likely to eliminate most of the spurious ‘events’ that were actually hospital transfers or readmissions for procedures following a previous event. We have also identified that a number of MI or other cardiac events may be miscoded as unspecified chest pain in the hospital admission record, suggesting that including this diagnosis when searching for cardiac events would increase the rate of detection, although possibly at the expense of a significant amount of additional case record review.

Events occurring outside of Scotland were a minor issue. Cross-linking with other nations within the UK would reduce this problem further. Events occurring outside the UK, other than deaths reported in UK death registries, or hospital cases transferred back to UK hospitals, would go undetected. However, the level of under-reporting present in the RL data was low and would be unlikely to have a substantial effect on the outcome of a trial.

The quality of matching of hospitalised stroke and TIA events was poorer since this type of event may be more likely to be misdiagnosed or miscoded. Nevertheless, RL picked up most of the WOSCOPS events, and the larger numbers of events that were recorded in the hospitalisations dataset than in WOSCOPS were mainly due to investigations or procedures for pre-existing conditions or previous events, issues that would be mostly resolved by case record review. It must also be borne in mind that stroke was not a specified endpoint in WOSCOPS and so we would expect a lower level of matching here due to a potentially less rigorous process of recording such events within the trial.

Clearly, in designing a clinical trial using RL data to detect clinical cardiovascular events, choice of endpoint would have to be made carefully. General cardiovascular or coronary hospitalisations are likely to be identified with high sensitivity and specificity. More focussed event types (such as MI or stroke) are likely to benefit from the use of RL for case identification followed by case note extraction and expert review.

In summary, we have shown that the wealth of routinely collected data could be used for cardiovascular outcome detection in a clinical trial carried out in a country with a unified health system, such as Scotland or the wider UK, and would likely lead to a qualitatively similar outcomes to those obtained by the current expensive clinical trial model involving rigorous individual patient follow-up and intensive data collection.

## Supporting Information

Appendix S1 **ICD 9 code groupings for each event type.**
(DOCX)
