# Supplementary material for: Are Routinely Collected NHS Administrative Records Suitable for Endpoint Identification in Clinical Trials? Evidence from the West of Scotland Coronary Prevention Study
Source: PLoS One. 2013 Sep 13;8(9):e75379. doi: 10.1371/journal.pone.0075379 (PMC3772901; doi:10.1371/journal.pone.0075379)
Supplement: Appendix S1 — ICD 9 code groupings for each event type. (DOCX) [file pone.0075379.s001.docx]

APPENDIX 1. ICD 9 code groupings for each event type

MI: 410

Other CHD: 411, 412, 413, 414

Other cardiac: 390, 391, 392, 393, 394, 395, 396, 397, 398, 401, 402, 403, 404, 405, 415, 416, 417, 420, 421, 422, 423, 424, 425, 426, 427, 428, 429, 435, 440, 442, 443, 444, 446, 447, 448, 451, 452, 453, 454, 455, 456, 457, 458, 459

Other vascular: 430, 431, 432, 433, 434, 436, 437, 438, 441

(Cardiovascular constitutes the codes of other cardiac and other vascular).

Unspecified chest pain: 7865

Stroke: 430, 431, 432, 433 (except 4331), 434, 436, 437 (except 4379), 438 (except 4389)

TIA: 435, 342
